# Multi-institutional validation of a radiomics signature for identification of postoperative progression of soft tissue sarcoma

**DOI:** 10.1186/s40644-024-00705-8

**Published:** 2024-05-08

**Authors:** Yuan Yu, Hongwei Guo, Meng Zhang, Feng Hou, Shifeng Yang, Chencui Huang, Lisha Duan, Hexiang Wang

**Affiliations:** 1 Department of Radiology, The Affiliated Hospital of Qingdao University, 16 Jiangsu Road, Qingdao, Shandong China; 2 Department of Operation Center, Women and Children’s Hospital, Qingdao University, Shandong, China; 3 Department of Pathology, The Affiliated Hospital of Qingdao University, Qingdao, Shandong China; 4 Department of Radiology, Shandong Provincial Hospital Affiliated to Shandong First Medical University, Jinan, Shandong China; 5 Department of Research Collaboration, Research and Development (R&D) center, Beijing Deepwise & League of Philosophy Doctor (PHD) Technology Co., Ltd, Beijing, China; 6 Department of Radiology, The Third Hospital of Hebei Medical University, Hebei, China

**Keywords:** Radiomics, Soft tissue sarcoma, Disease Progression, Progression-free survival

## Abstract

**Background:**

To develop a magnetic resonance imaging (MRI)-based radiomics signature for evaluating the risk of soft tissue sarcoma (STS) disease progression.

**Methods:**

We retrospectively enrolled 335 patients with STS (training, validation, and The Cancer Imaging Archive sets, *n* = 168, *n* = 123, and *n* = 44, respectively) who underwent surgical resection. Regions of interest were manually delineated using two MRI sequences. Among 12 machine learning-predicted signatures, the best signature was selected, and its prediction score was inputted into Cox regression analysis to build the radiomics signature. A nomogram was created by combining the radiomics signature with a clinical model constructed using MRI and clinical features. Progression-free survival was analyzed in all patients. We assessed performance and clinical utility of the models with reference to the time-dependent receiver operating characteristic curve, area under the curve, concordance index, integrated Brier score, decision curve analysis.

**Results:**

For the combined features subset, the minimum redundancy maximum relevance-least absolute shrinkage and selection operator regression algorithm + decision tree classifier had the best prediction performance. The radiomics signature based on the optimal machine learning-predicted signature, and built using Cox regression analysis, had greater prognostic capability and lower error than the nomogram and clinical model (concordance index, 0.758 and 0.812; area under the curve, 0.724 and 0.757; integrated Brier score, 0.080 and 0.143, in the validation and The Cancer Imaging Archive sets, respectively). The optimal cutoff was − 0.03 and cumulative risk rates were calculated.

**Data conclusion:**

To assess the risk of STS progression, the radiomics signature may have better prognostic power than a nomogram/clinical model.

**Supplementary Information:**

The online version contains supplementary material available at 10.1186/s40644-024-00705-8.

## Introduction

Soft tissue sarcoma (STS) is a highly aggressive and heterogeneous tumor with an overall 5-year survival rate of approximately 50% [1, 2]. Surgery is considered the standard treatment for localized STS. Despite appropriate aggressive multimodal therapy, the local recurrence rate is as high as 33–39% [3, 4], and approximately 25–30% patients have distant metastasis [5, 6]. Predicting progression and progression-free survival (PFS) in patients after surgical resection, to determine whether they should receive standard (or intensified) neoadjuvant chemoradiotherapy or neoadjuvant radiotherapy, could help delay disease progression and prolong survival [7]. Thus, developing STS-specific prognostic markers that can identify patient risk levels and aid treatment decision-making is warranted.

From a clinical perspective, prognostic and predictive models facilitate cancer management and treatment, personalized medicine, and forecasting of overall cancer outcomes [8]. At present, the tumor, node, metastasis system is limited in terms of anatomical tumor staging [9]. The French Federation of Cancer Centers Sarcoma Group (FNCLCC) and National Cancer Institute staging systems are important for obtaining prognostic models but rely on mitotic activity and necrosis to determine the final grade [2]. Moreover, the predictions of existing systems are not sufficiently accurate [9]. With rapid progress in our understanding of cancer biology, along with developments in medical imaging technology and new and effective therapies, investigators are now looking beyond current grading and staging systems and developing new predictive models.

Using high-dimensional medical images as a foundation, radiomics allows more sophisticated feature extraction than conventional visual interpretation [10]. By developing different models, radiomics has been used for predicting histopathological grade [11–13], risk of recurrence in cases of resection [14], preoperative lung metastasis status [15], and overall survival [16] in STS patients. However, although these models often include intra-tumoral (IT) STS lesions, analysis of the region surrounding the visible tumor (peri-tumoral [PT]) is lacking. Experimental evidence indicates that the microenvironment might have an integral role in STS tumor recurrence [17]. Therefore, it is critical to predict and assess radiomic signatures, which reflect microenvironmental invasion of the tumor periphery [18, 19]. We hypothesized that a radiomics signature based on IT and PT features would enhance the accuracy of STS prognostic predictions.

In this hypothesis-driven study, we assessed the performance of radiomics signatures based on IT, PT, and whole-tumoral (WT) features on magnetic resonance imaging (MRI) images for predicting the prognosis of STS patients. The signatures were developed using data from three institutions and The Cancer Imaging Archive (TCIA) database [20].

## Materials and methods

### Patients

This retrospective study received institutional review board approval, and the requirement for informed consent was waived for all participating institutions.

The training set consisted of 168 patients from the hospital 1. Two validation sets were created: the first set (validation set) was composed of 123 patients from two other hospitals (the hospital 2 and hospital 3), and the second set (TCIA set), used to assess generalizability, was generated using a publicly available dataset from TCIA (10.7937/K9/TCIA.2015.7GO2GSKS), from which we obtained a collection of images from 44 patients with STS [21].

Annex A1 supplements the criteria for inclusion and exclusion of patients.

Clinical data including age, gender, and FNCLCC grade were collected.

### Follow-up and survival analysis

All patients were followed up every 3–6 months with MRI or CT scanning during the 2 years following surgery and semiannually thereafter. Training and validation set data were censored in November 2021 and June 2020, respectively, and TCIA set data were censored in November 2011. PFS was defined as the time between surgery and radiographic detection of metastasis or recurrence, the day of death without evidence of progression, or the last negative follow-up.

### MRI semantic features acquisition

A total of 335 patients underwent preoperative T1-weighted imaging (T1WI) and fat-suppressed T2-weighted imaging (FS-T2WI). Supplementary A2 displays the inspection equipment information and Table S1 displays the MRI scan parameters.

After drawing on previous studies, we selected six features from the MRI semantic features (Supplementary A3).

### Tumor region delineation and radiomics feature extraction

**Fig. 1:**
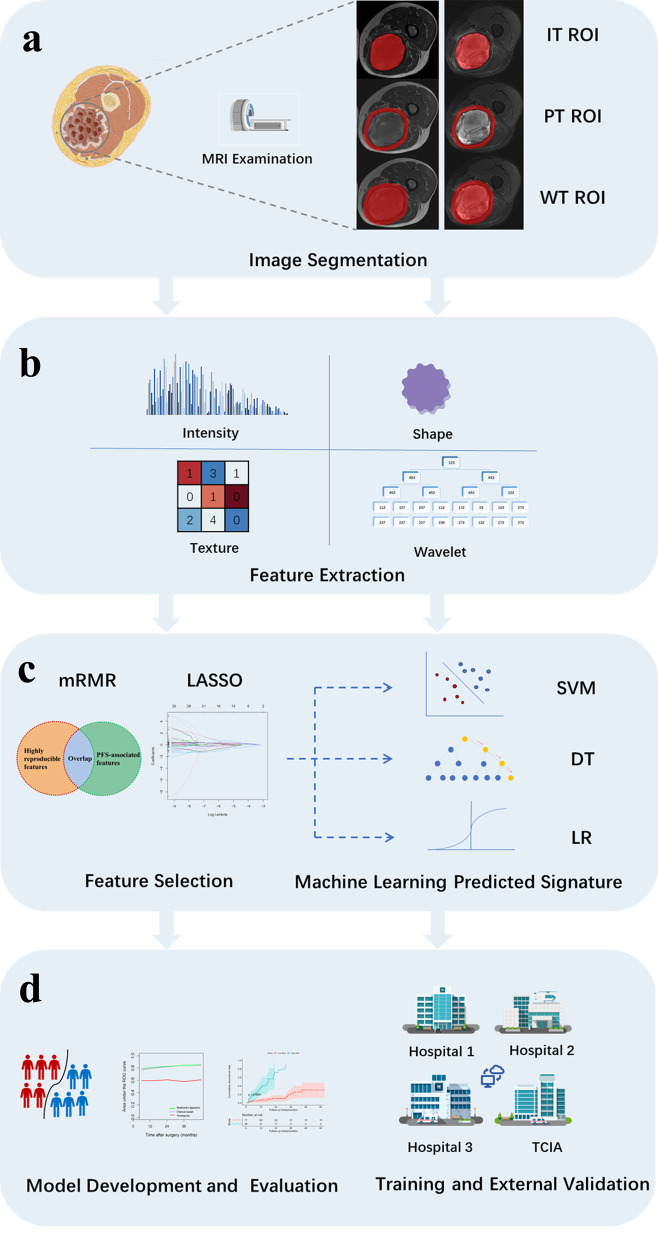
The study flow chart

The study flow chart is depicted in Fig. 1. ITK-SNAP (version 3.8.0; http://www.itksnap.org ) was employed to segment the region of interest (ROI) and evaluate the tumors in three dimensions. After segmentation, RIAS (version 0.2.1; https://pewter-papyrus-421.notion.site/RIAS-916ad7256e1e472985d4b11c8ebf0fe0) [22] was used to create peritumoral masks at a radial distance of 10 mm from the lesions in transverse and AP. Normal tissue, large arteries and veins, bronchi, and surrounding air were manually excluded. As Fig. 1(a) shows, the IT ROI corresponded to the maximum tumor area, the PT ROI to a radial distance of 10 mm from the lesion, and the WT ROI to the IT and PT regions combined.

Preprocessing procedures of features extraction were shown in Supplementary A4. Radiomics features were then extracted using 3D Slicer software (version 4.10.2; https://www.slicer.org/). Finally, radiomics features, including first-order statistical, shape-based, textural, and wavelet decomposition features, were extracted from each three-dimensional ROI of the FS-T2WI and T1WI sequences. Textural features were included five classes (gray-level run-length matrix gray-level run-length matrix, gray-level dependence matrix, gray-level cooccurrence matrix, and neighborhood gray-tone difference matrix). On the basis of the ROIs, the following features combination were created: (1) IT features, consisting of radiomics features in the IT ROIs of T1WI and FS-T2WI; (2) PT features, consisting of radiomics features in the PT ROIs of T1WI and FS-T2WI; (3) WT features, consisting of radiomics features in the WT ROIs of T1WI and FS-T2WI; and (4) Combined features, consisting of both IT and PT features.

The inter- and intraobserver performance of the radiomics feature extraction process was assessed by calculating intraclass correlation coefficients (ICCs). Images from 40 patients were randomly chosen for segmentation by multiple radiologists. Inter-observer correlation coefficients were calculated by manually segmenting ROIs, performed by Reader 1, and intraobserver correlation coefficients were calculated by repeating the segmentation after 1 month, performed by Reader 2. Features with an ICC of < 0.80 were removed because they were deemed to have poor agreement. Among them, 40 T1WI features and 66 T2WI features were removed in the IT features; 10 T1WI features and 56 T2WI features were removed in the PT features; 11 T1WI features and 7 T2WI features were removed in the WT features.

### “Combat compensation” method

The scanner effect is a major confounding factor in multi-center and multi-scheme studies that affects the extraction of radiomics features from MRI images [23]. Therefore, the combat compensation method was employed to eliminate the scanner effect.

### Construction of radiomics signature

**Fig. 2:**
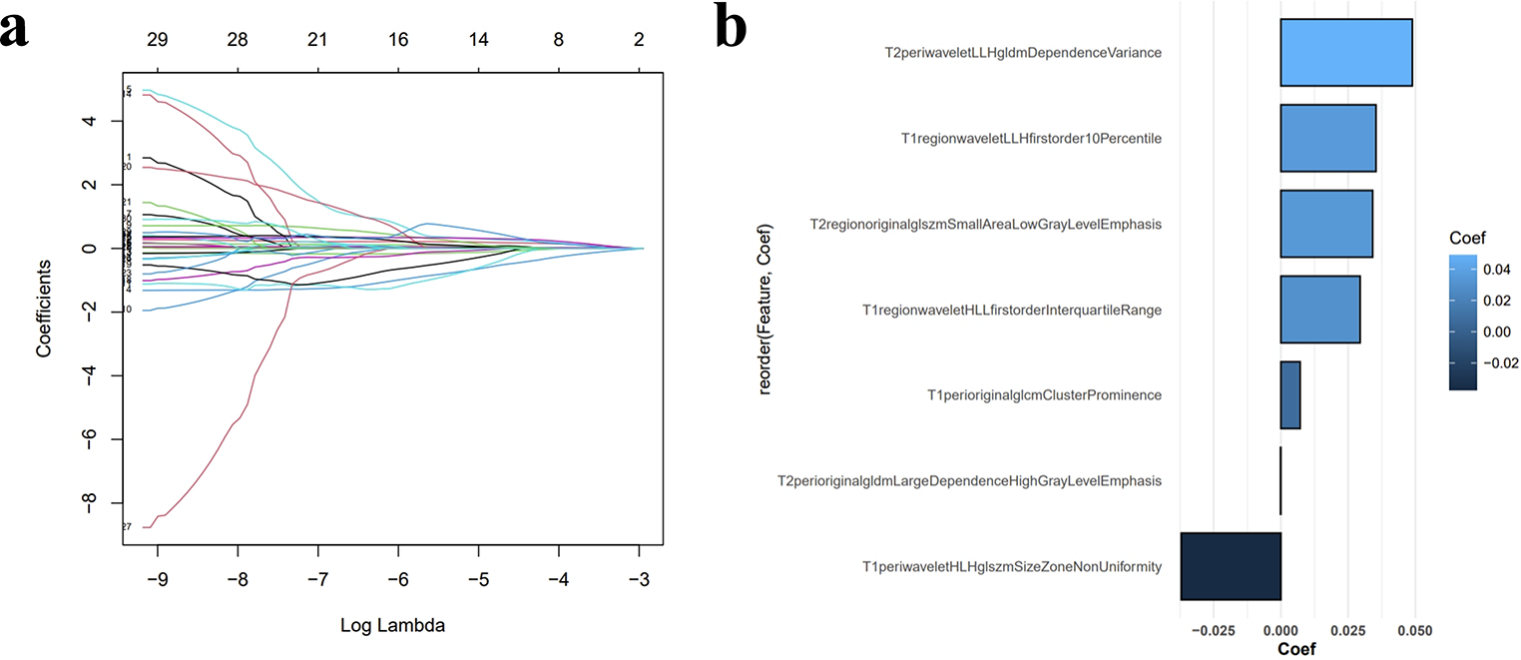
(**a**) MRI feature selection using the least absolute shrinkage and selection operator regression algorithm. (**b**) The seven selected MRI features and their coefficients

To remove the effect of varying gray values, all extracted radiomics features were normalized using z-scores. Because our feature pool had a high degree of dimensionality, feature selection was used to prevent overfitting. First, the 30 features with the strongest correlations and the least redundancy were selected by the minimum redundancy maximum relevance(mRMR) algorithm. Next, the feature parameters were further filtered using the least absolute shrinkage and selection operator (LASSO) regression algorithm (Fig. 2). Then, the following three machine learning classifiers were investigated: decision tree (DT), support vector machine (SVM), and logistic regression (LR). Three machine learning-predicted signatures were constructed for each feature subset, and a total of 12 machine learning-predicted signatures were built. Finally, the machine learning-predicted signature with the most accurate prediction results was selected, and its prediction score was inputted into Cox regression analysis to create the radiomics signature, which was used to obtain the radiomics score.

### Development of a clinical model and radiomics nomogram

Clinical information and MRI semantic features associated with STS progression were analyzed using univariate Cox regression, with factors significant at *p* < 0.05 considered significant independent risk factors for disease progression of STS patients. Such factor was included in the clinical model. Moreover, we integrated radiomics scores with selected clinical risk factors to develop a radiomics nomogram.

### Validation and performance evaluation of the different models

The prognostic performance of machine learning-predicted signatures was evaluated on the basis of area under the curve (AUC), accuracy, sensitivity, specificity, positive predictive, and negative predictive values. The ability of the clinical model, radiomics signature, and nomogram to predict the progression of STS patients was evaluated by the concordance index (C index) and the time-dependent receiver operating characteristic curve (T-ROC). The calibration curve was used to evaluate calibration ability. The integrated Brier score (IBS) was calculated using the “Boot632plus” splitting method to estimate the prediction error of the models. Decision curve analysis (DCA) was used to evaluate clinical usefulness. We used X-tile software (version 3.6.1; https://medicine.yale.edu/lab/rimm/research/software/) to identify optimal thresholds to classify patients into low- and high-risk groups on the basis of survival outcomes [24]. The Kaplan–Meier method and log-rank test were used to estimate the probability of PFS of the low- and high-risk groups.

### Statistics

The baseline data were compared using Fisher’s exact test, the chi-square test (for categorical variables) and the Mann-Whitney U test, Student’s t test (for continuous variables). SPSS (version 25.0; IBM Corp., Armonk, NY, USA) and R software (version 4.2.2; www.r-project.org) were used for the statistical analyses. Two-sided p-values < 0.05 indicated statistical significance.

## Results

### Clinical information and mri features of patients

Table S2 shows the diagnostic results and classifications of the 335 patients. The MRI morphological and clinical data of the patients are shown in Table S3. It can be seen that the PFS of the non-progression and progression groups differed significantly across two groups in three cohorts. In addition, gender, FNCLCC showed significant differences between the two groups with non-progression and progression groups in TCIA set. Both the training set and TCIA set showed significant differences in age. In univariable cox regression analysis, age was a significant independent predictor of STS progression (*p* < 0.05; Table 1). On the basis of these findings, the clinical model was established, and the AUC values for the training, validation, and TCIA sets were 0.593, 0.569, and 0.653, respectively.

**Table 1:** Univariable cox regression analysis of clinical and radiological features

		HR (95%CI)	*P* value
Clinical data	Age	1.014 (1.000–1.014)	0.037*
	Gender	1.069 (0.663–1.721)	0.784
Radiological Features	Number	0.991 (0.588–1.670)	0.974
	Depth	1.147 (0.713–1.841)	0.572
	Heterogeneous SI at T1WI	0.993 (0.610–1.616)	0.978
	Heterogeneous SI at T2WI	0.657 (0.404–1.068)	0.090
	Tumor volume with MRI signal compatible with necrosis	0.944 (0.661–1.345)	0.749
	Peritumoral edema	1.151 (0.756–1.752)	0.512

### Performance of the machine learning-predicted signatures

Table 2 displays the prediction performance of all machine learning-predicted signatures. In the Combined features subset, the mRMR-LASSO regression algorithm + DT classifier had the highest prediction accuracy, with AUC values of 0.812 (range: 0.730–0.893) and 0.856 (range: 0.750–0.963), and accuracy values of 0.789 and 0.795, in the validation and TCIA sets, respectively. Thus, the results for this algorithm were input into Cox regression analysis to construct the radiomics signature, which was then used to obtain the radiomics score.

**Table 2:** Performance of different machine learning algorithms in different features subsets

			Training set						Validation set						TCIA set					
**Features subsets**	**Classifiers**	AUC (95%CI)		ACC	SEN	SPE	PPV	NPV	AUC (95%CI)	ACC	SEN	SPE	PPV	NPV	AUC (95%CI)	ACC	SEN	SPE	PPV	NPV
Combined Features	LR	0.641(0.557–0.725)		0.607	0.289	0.828	0.540	0.625	0.555(0.452–0.658)	0.626	0.650	0.961	0.500	0.632	0.694(0.535–0.853)	0.659	0.833	0.450	0.645	0.692
	DT	0.865(0.806–0.925)		0.821	0.739	0.878	0.809	0.828	0.812(0.730–0.893)	0.789	0.717	0.831	0.718	0.831	0.856(0.750–0.963)	0.795	0.750	0.850	0.857	0.739
	SVM	0.621(0.536–0.705)		0.592	0.140	1.000	1.000	0.598	0.620(0.518–0.722)	0.626	0.000	1.000	NA	0.626	0.567(0.387–0.746)	0.590	1.000	0.100	0.571	1.000
IT Features	LR	0.679(0.596–0.762)		0.630	0.333	0.838	0.589	0.643	0.515(0.411–0.619)	0.512	0.195	0.701	0.281	0.593	0.588(0.500-0.763)	0.500	0.208	0.850	0.625	0.472
	DT	0.762(0.688–0.835)		0.738	0.565	0.858	0.735	0.739	0.536(0.436–0.636)	0.439	0.543	0.376	0.342	0.580	0.578(0.423–0.788)	0.431	0.541	0.300	0.481	0.352
	SVM	0.604(0.517–0.690)		0.601	0.028	1.000	1.000	0.596	0.505(0.397–0.612)	0.617	0.000	0.987	0.000	0.622	0.579(0.398–0.760)	0.431	0.00	0.950	0.00	0.441
PT Features	LR	0.624(0.537–0.710)		0.619	0.275	0.858	0.575	0.629	0.552(0.443–0.660)	0.520	0.347	0.623	0.355	0.615	0.550(0.372–0.728)	0.545	0.333	0.800	0.667	0.500
	DT	0.875(0.824–0.926)		0.797	0.840	0.707	0.716	0.873	0.571(0.471–0.672)	0.447	0.326	0.519	0.288	0.563	0.574(0.404–0.744)	0.477	0.375	0.600	0.529	0.400
	SVM	0.604(0.513–0.694)		0.595	0.014	1.000	1.000	0.592	0.567(0.458–0.676)	0.626	0.000	1.000	0.000	0.626	0.562(0.385–0.740)	0.450	0.000	1.000	0.000	0.450
WT Features	LR	0.646(0.563–0.729)		0.595	0.304	0.797	0.512	0.622	0.605(0.502–0.709)	0.537	0.087	0.805	0.211	0.596	0.515(0.335–0.694)	0.431	0.208	0.700	0.454	0.424
	DT	0.824 (0.760–0.887)		0.785	0.695	0.848	0.761	0.800	0.518(0.414–0.622)	0.471	0.326	0.558	0.306	0.581	0.475 (0.303–0.647)	0.500	0.458	0.550	0.550	0.458
	SVM			0.595	0.014	1.000	1.000	0.592	0.561 (0.452–0.671)	0.626	0.000	1.000	NA	0.626	0.523(0.346-0.700)	0.454	0.000	1.000	NA	0.454

*Note* AUC, area under curve; ACC, accuracy; SEN, sensitivity; SPE, specificity; PPV, positive predictive value; NPV, negative predictive value

### Construction of the nomogram and performance of the different models

**Fig. 3:**
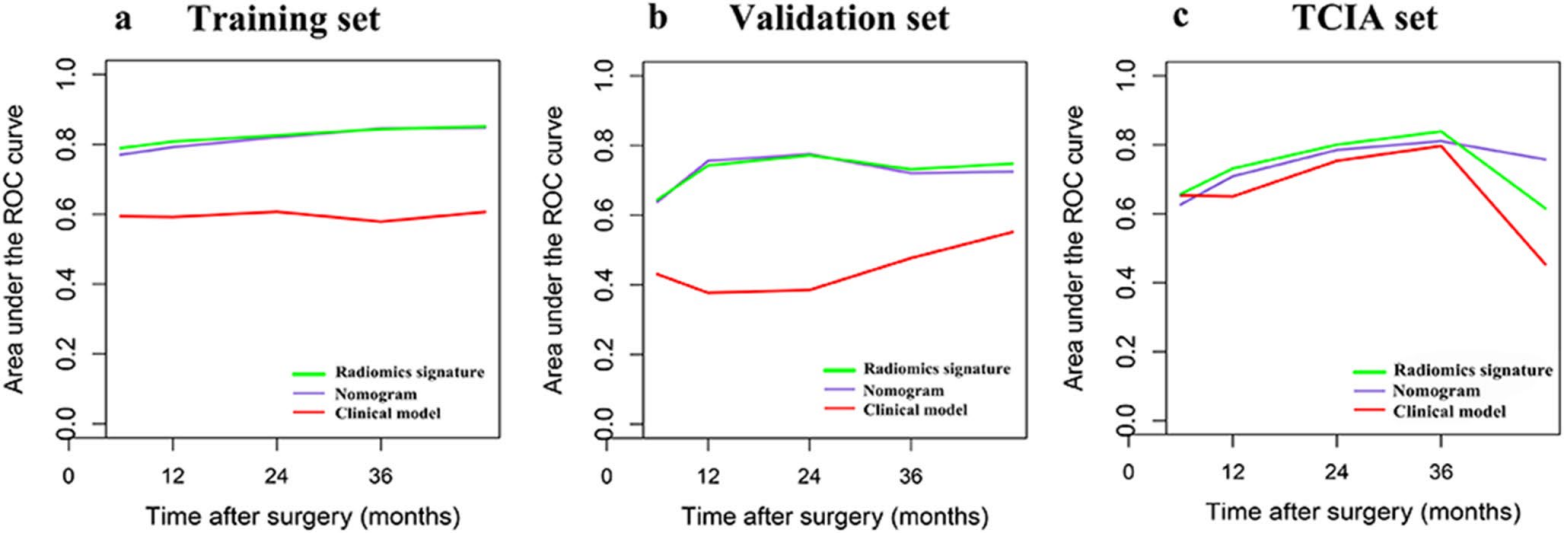
Time-dependent receiver operating characteristic curves for each model and the different cohorts

A nomogram was constructed by combining the radiomics signature with the clinical model derived from the univariate Cox regression analysis; Table 3 displays its predictive performance. The C index of the radiomics signature was 0.790 (range: 0.734–0.845) in the training set, 0.758 (range: 0.676–0.840) in the validation set, and 0.812 (0.683–0.941) in TCIA set, exceeding the values of other models. According to the T-ROC, the AUC values of the radiomics signature were similar to those of the nomogram, and both were higher than those of the clinical model (Fig. 3).

**Table 3:** Predictive performance of radiomics signature, nomogram and clinical model

	Training set			Validation set			TCIA set			
	AUC	C index(95%CI)	IBS	AUC	C index(95%CI)	IBS	AUC	C index(95%CI)	IBS	
Radiomics signature	0.820	0.790(0.734-0.845)	0.112	0.724	0.758(0.676-0.840)	0.080	0.757	0.812(0.683-0.941)	0.143	
Nomogram	0.776	0.776(0.725-0.828)	0.111	0.742	0.735(0.664-0.805)	0.082	0.730	0.707(0.592-0.821)	0.143	
Clinical model	0.593	0.583(0.512-0.655)	0.128	0.569	0.551(0.460-0.642)	0.085	0.653	0.644(0.520-0.767)	0.155	

*Note* AUC, area under curve; C index, concordance index; IBS, integrated Brier score

**Fig. 4:**
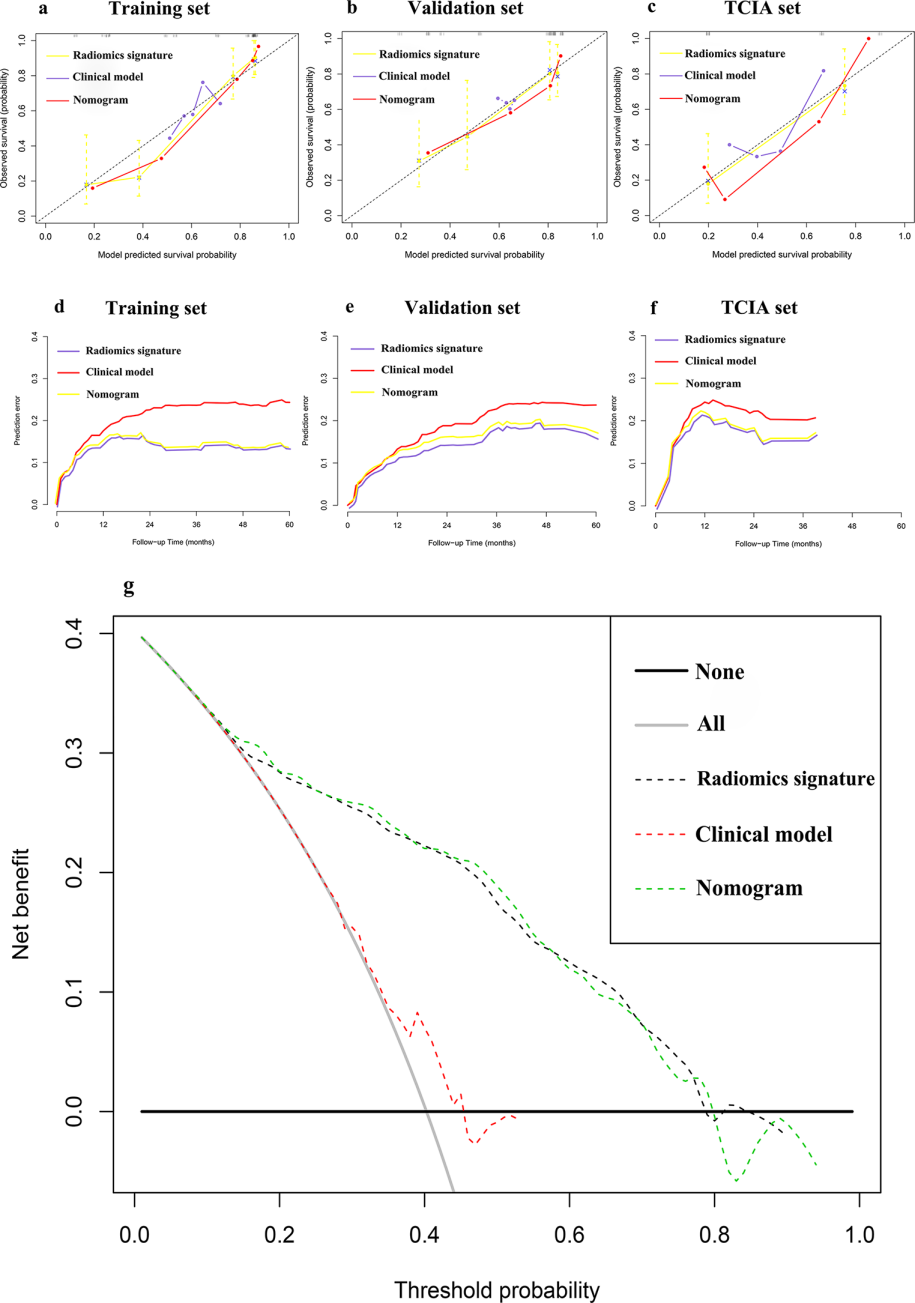
(**a–c**) Calibration curves of the different models using the training, validation, and TCIA sets. (**d–f**) Prediction error curves of the different models using the training, validation, and TCIA sets. (**g**) Results of the decision curve analysis of all cohorts

Figure 4(a–c) shows calibration plots of the different models predicting STS progression over 3 years. Figure 4(d–f) shows the prediction error of the different models. The IBS values of the radiomics signature, nomogram, and clinical model were 0.080, 0.082, and 0.085 in the validation set and 0.143, 0.143, and 0.155 in TCIA set, respectively. Therefore, the radiomics signature had good calibration ability and less prediction error than the other models. In addition, as shown in Fig. 4(g), the radiomics signature provided the greatest clinical benefit according to 3-year DCA.

### Risk stratification

**Fig. 5:**
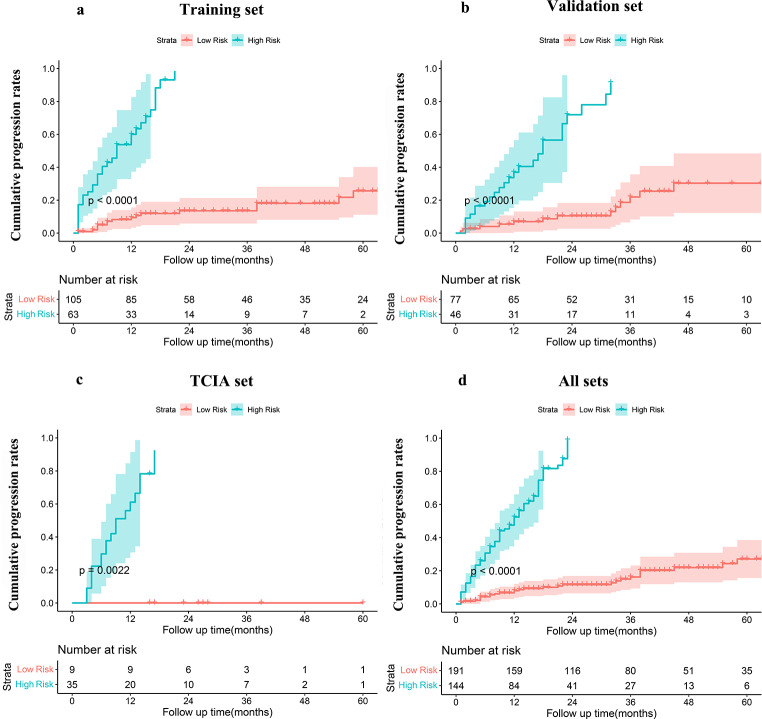
Cumulative progression rates predicted by the radiomics signature according to risk group

We established two STS progression risk groups according to a cutoff value of -0.03. Table 4 lists the median PFS times of the different cohorts, as well as the cumulative 2-, 3-, and 5-year PFS rates. Cumulative progression rates are presented in Fig. 5.

**Table 4:** The median PFS and cumulative 2-, 3-, and 5-year PFS rates in different cohorts

Cohort	No. of patients (progression numbers)	Median PFS (months)	2-year PFS rate (%)	3-year PFS rate (%)	5-year PFS rate (%)	*P* value
**Training Set**						
Low risk	105(18)	NA(NA-NA)	0.873(0.808-0.944)	0.834(0.755-0.922)	0.774(0.669-0.894)	
High risk	63(51)	15(9-23)	0.270(0.173-0.421)	0.204(0.118-0.352)	0.051(0.014-0.190)	*p<0.001
**Validation Set**						
Low risk	77(13)	NA(NA-NA)	0.900(0.833-0.974)	0.803(0.700-0.921)	0.738 (NA-NA)	
High risk	46(33)	23(17-37)	0.487(0.356-0.666)	0.301(0.182-0.498)	0.089(0.025-0.314)	*p<0.001
**TCIA set**						
Low risk	9(0)	NA (NA-NA)	NA (NA-NA)	NA (NA-NA)	NA (NA-NA)	
High risk	35(24)	14(9-NA)	0.325(0.198-0.535)	0.271(0.147-0.500)	NA (NA-NA)	*p=0.002
**All sets**						
Low risk	191(31)	NA(NA-NA)	0.890(0.845-0.939)	0.850(0.793-0.911)	0.763(0.680-0.855)	
High risk	144(108)	17(13-23)	0.363(0.289-0.457)	0.264(0.195-0.358)	0.084(0.039-0.181)	*p<0.001

Note PFS, progression-free survival; *, In comparison to the low-risk stratification

As shown by the cumulative progression curves, the radiomics signature significantly stratified patients according to STS risk in the training, validation, TCIA sets, and all sets combined (all log-rank p-values < 0.01). The median PFS times of the low-risk groups were not reached in all cohorts, and the median PFS times of the high-risk groups were 15, 23, 14, and 17 months in the training set, validation set, TCIA set, and all sets combined, respectively.

## Discussion

The use of radiomics in preoperative prognostic models could facilitate risk stratification of STS patients [14–16]. However, the potential of PT images, which provide prognostically relevant information that could aid prediction of STS progression, remains to be explored. Moreover, prognostic models have not been evaluated in multi-institutional, international cohorts (including TCIA database). On the basis of the radiomics features in the ROIs in this study, data from 335 STS patients were used to construct 12 machine learning-predicted signatures for identifying postoperative progression of STS. Combining multidimensional IT and PT features enhanced the accuracy of STS risk stratification. Furthermore, compared with the clinical model and nomogram, the radiomics signature had greater prognostic capacity (AUC = 0.820, 0.724, and 0.757 using the training, validation, and TCIA sets, respectively), superior clinical value, good calibration ability, and low prediction error (IBS ≤ 0.143). Moreover, the radiomics signature performed similarly across all three cohorts, indicating universality and stability.

MRI has been applied for assessing the prognosis of STS [25]. According to Amandine et al., overall survival in STS may be associated with certain MRI features, including a heterogeneous T2WI signal, peritumoral enhancement, and necrosis [26]. In this study, the above MRI features were not meaningful, which reflects model instability. Moreover, our clinical model included only age and showed poor differentiation performance. Compared with previously reported predictors, the radiomics features in this study had lower prediction error and superior clinical value, indicating that the radiomics signature is the most valid and dependable method for predicting STS prognosis.

Radiomics, as an emerging non-invasive method, mines quantitative features in medical images to obtain markers that help clinicians make clinical decisions [27, 28]. Radiomics can be regarded as a “digital biopsy” that allows in-depth analysis of spatial heterogeneity and tumor phenotypes in various clinical scenarios [29, 30]. Because of the aggressive nature of cancer, the peritumoral region provides useful and complementary information about the disease. The survival likelihood of cancer patients is impacted by tumor invasion into the peripheral region [31]. Previous research described the application of radiomic techniques to capture information about areas surrounding sites of cancer [31, 32]. Sun et al. [33] combined 5-mm IT and PT regions to predict axillary lymph node metastasis in breast cancer. Braman et al. [34] incorporated features from 6–12-mm PT regions to characterize the so-called “response-associated HER2-E subtype.” In our study, when we defined the PT region as a 15-mm area outside of the lesions, normal tissue and air covering a large area had to be manually excluded in some cases. In view of the above, 10-mm peritumoral masks at a radial distance of 10 mm from the lesions were generated in this study. The results showed that the performance achieved by combining the IT and PT regions was significantly improved, similar to previous results [33, 35].

Previous studies have shown that radiomics features can predict survival outcomes in STS patients. Liang et al. [15] developed a radiomics nomogram based on FS-T2WI and T1WI sequences that provided satisfactory PFS risk stratification for STS patients. A radiomics signature based on FS-T2WI also achieved good prognostic results in terms of risk stratification for overall survival [12]. The radiomics features that we used to predict cumulative progression were satisfactory for risk stratification of STS patients. Neoadjuvant radiotherapy and chemotherapy could lower the risk of disease progression and improve the survival of STS patients [7, 36], and more aggressive treatment should be implemented as early as possible for patients at high risk of disease progression. Currently, developing individualized treatment plans and choosing appropriate postoperative follow-up times for STS patients is a major challenge. In our study, STS patients were categorized into low- and high-risk groups on the basis of the radiomics signature. For the low-risk group, the 2-, 3-, and 5-year cumulative progression rates were 11.0%, 15.0%, 23.7%, respectively, in all sets. Therefore, complete surgical resection and routine follow-up after surgery are recommended, whereas adjuvant treatment is generally not appropriate after surgery. For the high-risk group, the 2-, 3-, and 5-year cumulative progression rates were 63.7%, 73.6%, and 91.6%, respectively, in all sets. Therefore, standard-dose adjuvant chemoradiotherapy after surgery is recommended, along with appropriate targeted therapy or immunotherapy regimens and close follow-up after surgery.

Our study differs from some previously published STS radiomics studies in several ways. First, it is the first study to show the added value of PT features for STS risk stratification when combined with IT features. Second, it is the first study to assess the role of radiomics in STS patients using TCIA database, which includes patients from various geographic regions, as well as different MRI scanners and imaging protocols. Third, we extracted radiomics features using conventional T1WI and FS-T2WI sequences, which are clinically reproducible, readily available, widely used, and more familiar to radiologists compared with dynamic contrast-enhanced imaging.

The current study had some limitations. First, given its retrospective nature, and although we used rigorous criteria, selection bias cannot be ruled out. Second, because the robustness and generalizability of radiomics across multiple institutions, MRI scanners, and parameter settings is important, we verified our radiomics nomogram by adding an open-source data, i.e., TCIA database. However, TCIA sample size is small, and larger prospective samples are required for further verification. Third, the stability of the radiomics features may be affected when segmentation is performed by multiple radiologists. Even if we exclude features with ICC values < 0.80, the efficiency of the radiomics process could be improved by more accurate automated tumor segmentation [37]. Finally, a radial distance of 10 mm from the lesion was considered the peritumor region, and the distension area around the lesion will be further explored in a future study.

## Conclusion

In summary, we proposed a radiomics signature based on heterogeneous IT and PT features that could serve as a non-invasive and accessible biomarker to effectively predict outcomes and add prognostic value to traditional radiomics signatures based only on IT regions, thus promoting precise transplant oncology and medical imaging.

### Electronic supplementary material

Below is the link to the electronic supplementary material.

Supplementary Material 1

Supplementary Material 2

Supplementary Material 3

## Data Availability

The datasets used and/or analysed during the current study are available from the corresponding author on reasonable request.

## Abbreviations

MRI: magnetic resonance imaging
STS: soft tissue sarcoma
PFS: progression-free survival
FNCLCC: The French Federation of Cancer Centers Sarcoma Group
IT: intra-tumoral
PT: peri-tumoral
WT: whole-tumoral
TCIA: The Cancer Imaging Archive
T1WI: T1-weighted imaging
FS-T2WI: fat-suppressed T2-weighted imaging
ROI: region of interest
ICCs: intraclass correlation coefficients
mRMR: the minimum redundancy maximum relevance
LASSO: the least absolute shrinkage and selection operator
DT: decision tree
SVM: support vector machine
LR: logistic regression
AUC: the basis of area under the curve
C index: the concordance index
T-ROC: the time-dependent receiver operating characteristic curve
IBS: The integrated Brier score
DCA: Decision curve analysis
