# Supplementary material for: Multi-institutional validation of a radiomics signature for identification of postoperative progression of soft tissue sarcoma
Source: Cancer Imaging. 2024 May 8;24:59. doi: 10.1186/s40644-024-00705-8 (PMC11077743; doi:10.1186/s40644-024-00705-8)
Supplement: Supplementary file 1 — Supplementary Material 1 [file 40644_2024_705_MOESM1_ESM.docx]

**Supplementary material**

**Supplementary A1: The inclusion and exclusion criteria of patients**

Preoperative MRI data of STS patients in the training set from May 2008 and November 2021, and in the validation set from November 2007 and June 2020, were analyzed. The inclusion criteria were as follows: 1) availability of clinical data and pathological confirmation; and 2) MRI examination conducted within 2 weeks before surgery. The exclusion criteria were: 1) an image signal-to-noise ratio < 1.0; 2) presence of other, unrelated malignant tumors; and 3) incomplete follow-up information.

Patients in TCIA set from November 2004 and November 2011 met the following criteria: 1) histologically proven primary STS of the extremities; 2) no metastatic and/or recurrent STS at presentation; 3) no lung metastasis (among patients who were followed up for < 12 months).

**Supplementary A2: The inspection equipment information**

The MRI scanners used to acquire the training and validation set data were as follows: 1.5-T and 3.0-T GE HDx (GE HealthCare, Chicago, IL, USA), 3.0-T Magnetom Skyra and Prisma (Siemens Healthineers, Erlangen, Germany), and 1.5-T Philips Achieva (Philips Healthcare, Amsterdam, Netherlands). Table 1 displays the MRI scan parameters.

**Supplementary A3:** **The MRI semantic features acquisition**

The MRI semantic features were evaluated by three radiologists who had been engaged in musculoskeletal system diagnosis for > 3 years. The semantic features included: 1) number (solitary or multiple); 2) depth (deep or superficial; maximum lesion depth ≥ 8 cm was defined as deep); 3) tumor volume showing an MRI signal indicative of necrosis (0%, 1%–50%, or ≥ 50% of the tumor volume); 4) heterogeneous signal intensity on FS-T2WI (< 50% or ≥ 50%); 5) heterogeneous signal intensity on T1WI (< 50% or ≥ 50%); and 6) peritumoral edema (without, with limited, or with extensive peritumoral edema). Semantic features were acquired from the main part of the lesion. If a dispute arose during feature analysis, all three radiologists reviewed the images together and reached a consensus.

**Supplementary A4: Preprocessing procedures of features extraction**

To reduce feature variability, preprocessing was performed to compensate for inhomogeneous intensity resulting from the use of different protocols and field strengths. Gray-level quantization reduced the number of gray levels and improved the signal-to-noise ratio of the texture computations. The voxel spacing was standardized using cubic interpolation to resample the three-dimensional ROIs to isotropic resolution (voxel size = 1 × 1 × 1 mm^3^) (1,2).

**Table S1 Magnetic Resonance Parameters in the Training Set and Validation Set**

|  | T1WI | FS-T2WI |
| --- | --- | --- |
| Repetition time(ms) | 500–600 | 2400–4500 |
| Echo time(ms) | 10–15 | 70–120 |
| Field of view(mm) | 200–400 mm | 200–400 mm |
| Matrix | 320 × 320 | 320 × 320 |
| Section thickness(mm) | 3–5 mm | 3–5 mm |
| Section spacing(mm) | 1 | 1 |

| **Table S2 Summary of 335 soft-tissue sarcoma patients confirmed by pathologic results** | | | |  |
| --- | --- | --- | --- | --- |
|  | **Training Set**  **(n=168)** | **Validation Set**  **(n=123)** | **TCIA set**  **(n=44)** | |
| Pleomorphic sarcoma, undifferentiated | 23 | 13 | 5 | |
| Malignant peripheral nerve sheath rumour | 3 | 1 | 0 | |
| Extraskeletal osteosarcoma | 6 | 1 | 3 | |
| Extraskeletal myxoid chondrosacamo | 3 | 3 | 0 | |
| Rhabdomyosarcoma | 4 | 13 | 1 | |
| Synovial sarcoma | 10 | 15 | 5 | |
| Kaposi sarcoma | 1 | 0 | 0 | |
| Leiomyosarcoma | 13 | 9 | 9 | |
| Epithelioid sarcoma | 1 | 2 | 2 | |
| Spindle cell sarcoma, undifferentiated | 4 | 7 | 1 | |
| Undifferentiated sarcoma | 17 | 3 | 1 | |
| Myxofibrosarcoma | 29 | 20 | 5 | |
| Alveolar soft part sarcoma | 4 | 3 | 0 | |
| Angiosarcoma | 1 | 3 | 0 | |
| Solitary fibrous tumour, malignant | 1 | 3 | 0 | |
| Liposarcoma | 35 | 23 | 11 | |
| Others | 13 | 4 | 1 | |
| Note: TCIA: The Cancer Imaging Archive |  |  |  | |

| **Table S3 Patient and Tumor Characteristics** | | | | | | | | | |
| --- | --- | --- | --- | --- | --- | --- | --- | --- | --- |
|  | **Training Set (N=168)** | | | **Validation Set (N=123)** | | | **TCIA Set (N=44)** | | |
|  | **Non-progression**  **(N=99)** | **Progression**  **(N=69)** | ***p*** | **Non-progression**  **(N=77)** | **Progression**  **(N=46)** | ***p*** | **Non-progression**  **(N=20)** | **Progression**  **(N=24)** | ***p*** |
| Age(years)  (mean±SD) | 49.9±20.0 | 55.8±17.1 | 0.048 | 50.6±18.6 | 50.9±16.7 | 0.971 | 47.4±22.3 | 58.9±9.78 | 0.041 |
| Gender |  |  |  |  |  |  |  |  |  |
| Male | 43 | 30 | 1.000 | 32 | 15 | 0.323 | 14 | 9 | 0.040 |
| Female | 56 | 39 |  | 45 | 31 |  | 6 | 15 |  |
| FNCLCC |  |  |  |  |  |  |  |  |  |
| I | 19 | 12 | 0.895 | 25 | 12 | 0.451 | 5 | 5 | 0.030 |
| II | 35 | 27 |  | 29 | 15 |  | 6 | 14 |  |
| III | 45 | 30 |  | 23 | 19 |  | 9 | 25 |  |
| PFS | 42.2±36.9 | 17.8±19.6 | ＜0.001 | 35.5±21.8 | 20.9±16.1 | ＜0.001 | 34.2±15.5 | 10.8±8.51 | ＜0.001 |
| Heterogeneous SI at T1WI |  |  |  |  |  |  |  |  |  |
| (+) | 38 | 26 | 1.000 | 39 | 22 | 0.853 | 1 | 3 | 0.641 |
| (-) | 61 | 43 |  | 38 | 24 |  | 19 | 21 |  |
| Heterogeneous SI at T2WI |  |  |  |  |  |  |  |  |  |
| (+) | 46 | 27 | 0.429 | 45 | 23 | 0.454 | 6 | 13 | 0.135 |
| (-) | 53 | 42 |  | 32 | 23 |  | 14 | 11 |  |
| Number |  |  |  |  |  |  |  |  |  |
| Solitary | 75 | 49 | 0.593 | 57 | 30 | 0.299 | 19 | 23 | 1.000 |
| Multiple | 24 | 20 |  | 20 | 16 |  | 1 | 1 |  |
| Depth |  |  |  |  |  |  |  |  |  |
| Deep | 48 | 37 | 0.534 | 41 | 23 | 0.852 | 11 | 9 | 0.363 |
| Superficial | 51 | 32 |  | 36 | 23 |  | 9 | 15 |  |
| Necrosis |  |  |  |  |  |  |  |  |  |
| 0 | 26 | 20 | 0.821 | 16 | 7 | 0.704 | 11 | 5 | 0.061 |
| 1%–50% | 49 | 35 |  | 46 | 28 |  | 8 | 16 |  |
| >50% | 24 | 14 |  | 15 | 11 |  | 1 | 3 |  |
| Peritumoral edema |  |  |  |  |  |  |  |  |  |
| No | 24 | 14 | 0.777 | 19 | 11 | 0.568 | 7 | 5 | 0.057 |
| Limited | 63 | 45 |  | 51 | 28 |  | 10 | 19 |  |
| Extensive | 12 | 10 |  | 7 | 7 |  | 3 | 0 |  |
| Note: TCIA，The Cancer Imaging Archive; FNCLCC，French Federation Nationale des Centres de Lutte Contre le Cancer; PFS，progression-free survival | | | | | | | | | |

**References**

1. Depeursinge A, Foncubierta-Rodriguez A, Van De Ville D, Müller H. Three-dimensional solid texture analysis in biomedical imaging: review and opportunities. Medical image analysis 2014;18(1):176-196.

2. Gibbs P, Turnbull LW. Textural analysis of contrast-enhanced MR images of the breast. Magnetic resonance in medicine 2003;50(1):92-98.
