# Supplementary material for: Multi-institutional validation of a radiomics signature for identification of postoperative progression of soft tissue sarcoma
Source: Cancer Imaging. 2024 May 8;24:59. doi: 10.1186/s40644-024-00705-8 (PMC11077743; doi:10.1186/s40644-024-00705-8)
Supplement: Supplementary file 2 — Supplementary Material 2 [file 40644_2024_705_MOESM2_ESM.docx]

**Revision Notes**

Dear Editors,

Thank you for your letter and for the reviewer’s comments concerning our manuscriptentitled " Multi-institutional Validation of a Radiomics Signature for Identification of Postoperative Progression of Soft Tissue Sarcoma" CAIG-D-23-00753.

We have studied comments carefully and the responds to the reviewer’s comments are described below.

Thanks again for your time and letter.

**Reviewer1**

**Q1: About the definitions of IT, PT and WT, please explain why PT is included in WT(Fig1), WT is not the IT?**


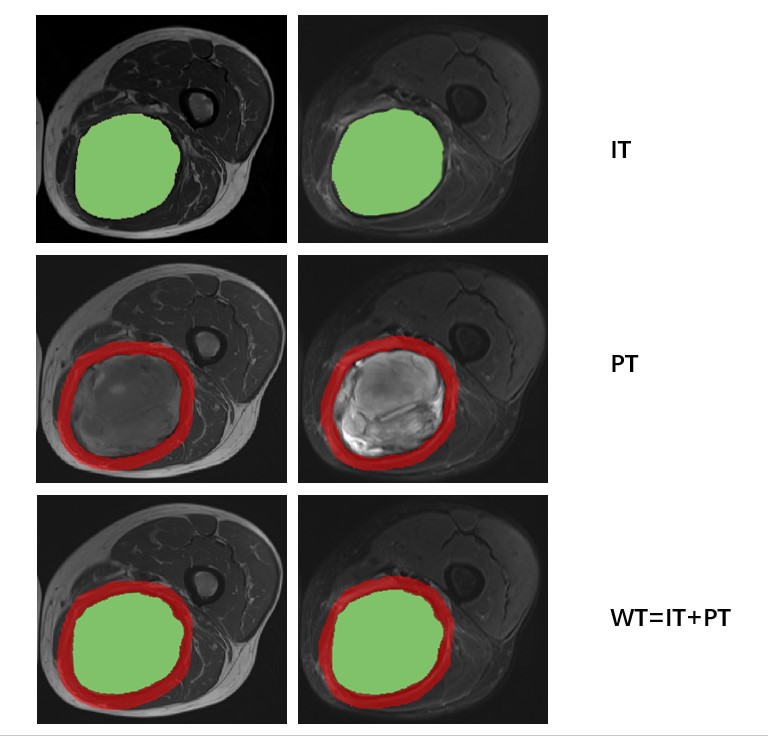
**[Response]** Thanks for your question. WT ROIs=IT ROIs + PT ROIs. As schema shows, the IT ROI corresponded to the maximum tumor area, the PT ROI to a radial distance of 10 mm from the lesion, and the WT ROI to the IT and PT regions combined.

**Q2:** **About the inclusion and exclusion criteria of patients: Preoperative MRI data of STS patients in the training set from May 2008 and November 2021, and in the validation set from November 2007 and June 2020, were analyzed. While Table 4 (The median PFS and cumulative 2-, 3-, and 5-year PFS rates in different cohorts) shows risk stratification, but how to calculate 5-year PSF for patients in the years of 2021and 2020?**

**[Response]** Thanks for your question. Training set data were censored in November 2021, validation set data were censored in June 2020. PFS was defined as the time between surgery and radiographic detection of metastasis or recurrence, the day of death without evidence of progression, or the last negative follow-up.

PFS = date of endpoint event (disease progression or death) - start date + 1;

PFS = date of censored event - start date + 1;

Depending on the occurrence of survival outcomes, data for survival analyses are often divided into two categories: endpoint events and censored. If the endpoint event has not occurred by the end date of the study, we refer to this type of data as censored data, and the Kaplan-Meier method makes full use of the incomplete information provided by censored data in calculating survival rates.

The Kaplan-Meier method, also known as the Product-limit Estimate, is one of the most commonly used survival analysis methods. At the beginning of the study, i.e., when the time is 0, the survival rate is 1, i.e., S(0)=1. When the observation time comes to the node of the 1st month, there are a total of 168 people in the training set who are at risk for progression before this time node, and eventually 11 cases of endpoint events have occurred, and the probability of survival at this point of time is 1-(11/168)=0.935, and the probability calculated at this point is actually the conditional probability of the patient at the previous time point without progression, then the survival rate at this time point should be expressed as S(1) = 0.935*S(0), and so on, and the survival rate at each time point, i.e., the cumulative survival probability, can be obtained by multiplying the survival probability at that time point by the survival rate at the previous time point.

It is important to note that when there is censoring, survival at this time point does not change, but the number of censored individuals needs to be subtracted when calculating the number of individuals at risk of progression at the next time point. For example, if a patient has reached the follow-up cutoff date but has not experienced an endpoint event by month 24, the probability of survival at this time point is calculated as described above, whereas at month 25, this patient is no longer included in the calculation of subsequent progression-free survival as censored data.

**Q3: Table S2 Summary of 335 soft-tissue sarcoma patients confirmed by pathologic results, there are many STS types, please explain the influence of different pathologic types on the results.**

**[Response]** Thank you for your comments. It is true that different types lead to different biological behaviors, but we do not have experience of exactly how much of an impact this will have, and we will add relevant predictive studies of specific pathological types of soft tissue sarcoma to our follow-up studies. At the same time, limited by case numbers, mainstream studies now include multiple pathological types of soft tissue sarcomas for clinical investigation [1-6], and this approach was used in the present study to investigate the same situation.

1. Crombé A, Marcellin PJ, Buy X, Stoeckle E, Brouste V, Italiano A, Le Loarer F, Kind M. Soft-Tissue Sarcomas: Assessment of MRI Features Correlating with Histologic Grade and Patient Outcome. Radiology. 2019 Jun; 291(3):710-721.

2. Peeken JC, Spraker MB, Knebel C, Dapper H, Pfeiffer D, Devecka M, Thamer A, Shouman MA, Ott A, von Eisenhart-Rothe R, Nüsslin F, Mayr NA, Nyflot MJ, Combs SE. Tumor grading of soft tissue sarcomas using MRI-based radiomics. EBioMedicine. 2019 Oct; 48:332-340.

3. Zhao F, Ahlawat S, Farahani SJ, Weber KL, Montgomery EA, Carrino JA, Fayad LM. Can MR imaging be used to predict tumor grade in soft-tissue sarcoma? Radiology. 2014 Jul; 272(1):192-201.

4. Liu S, Sun W, Yang S, Duan L, Huang C, Xu J, Hou F, Hao D, Yu T, Wang H. Deep learning radiomic nomogram to predict recurrence in soft tissue sarcoma: a multi-institutional study. Eur Radiol. 2022 Feb; 32(2):793-805.

5. Wang H, Chen H, Duan S, Hao D, Liu J. Radiomics and Machine Learning With Multiparametric Preoperative MRI May Accurately Predict the Histopathological Grades of Soft Tissue Sarcomas. J Magn Reson Imaging. 2020 Mar; 51(3):791-797.

6. Yan R, Hao D, Li J, Liu J, Hou F, Chen H, Duan L, Huang C, Wang H, Yu T. Magnetic Resonance Imaging-Based Radiomics Nomogram for Prediction of the Histopathological Grade of Soft Tissue Sarcomas: A Two-Center Study. J Magn Reson Imaging. 2021 Jun; 53(6):1683-1696.

**Q4:** **Please explain how to caculate tumor volume with MRI signal compatible with necrosis in the study?**

**[Response]** Thanks for your question. Tumor volume with MRI signal compatible with necrosis were assessed on T2WI sequence as 0, 1%-50%, >50%: (1) 0: 0% of the high signal at T2-weighted imaging; (2) 1%-50%: less than 50% of the high signal at T2-weighted imaging;(3)＞50%: at least 50% of the high signal at T2-weighted imaging.

A threshold value of 0 indicates the presence or absence of necrotic areas, which can be easily determined on the image, while most lesions can be identified by visual judgement if the degree of necrosis is >50%. However, for cases extremely close to 50%, we used PACS to measure and calculate the volume of the necrotic area, which was compared with the tumor volume, and the methods were obtained in the following way.

The tumor volume was independently calculated by two radiologists. The maximal intramedullary extension of each lesion was measured from the coronal plane on T1WI, while the widths and depths were measured from the axial plane on FS-T2WI. When these two reviewers found more than 10% discrepancy in tumor volume, the tumors were remeasured, and agreement was reached by consultation. The tumor volume was then calculated using the standard mathematical formula for an ellipsoid (0.52×length ×width×depth) [1]. Then, the length, width and depth of the necrotic area are measured on FS-T2WI and calculated in the same way. Finally, the ratio of necrotic volume to tumor volume was calculated to determine whether the necrotic volume was >50%.

1. Bieling P, Rehan N, Winkler P, Helmke K, Maas R, Fuchs N, et al. Tumor size and prognosis in aggressively treated osteosarcoma. J Clin Oncol 1996;14:848-858

**Reviewer2**

**Q1:** **For the peritumoral masks, was the radial distance of 10 mm in all axes including craniocaudal, transverse, and AP?**

**[Response]** Thanks for your question. For the peritumoral masks, was the radial distance of 10 mm in transverse and AP, but not in craniocaudal. In addition, Normal tissue, large arteries and veins, bronchi, surrounding air were manually excluded.

**Q2:** **It states "Features with an ICC of <0.80 were removed because they were deemed to have poor agreement." How many features were removed?**

**[Response]** Thanks for your question. 40 T1WI features and 66 T2WI features were removed in the IT features; 10 T1WI features and 56 T2WI features were removed in the PT features; 11 T1WI features and 7 T2WI features were removed in the WT features.

**Q3:** **The concordance index of the radiomics signature was higher than other methods, but the confidence intervals overlap. Is this considered a significant difference for concordance indices?**

**[Response]** Thanks for your question. We used the anova function in R to compare the concordance index of Radiomics signature, Nomogram, and Clinical model, with a statistical result of P＜0.05. Given this, we consider the concordance index to be statistically different, and the overlap of confidence intervals may not be directly related to the absence of statistical significance. Also, the AUC of Radiomics signature, Nomogram, and Clinical model, were differentiated by the Delong test.

**Q4:** **Why weren't routine contrast-enhanced imaging sequences used?**

**[Response]** Thanks for pointing out the problem. Because contrast-enhanced imaging sequences were not used in the TCIA database, in order to include these data, contrast-enhanced imaging sequences were not used in this study. In addition, there were only 162 patients that we merged with contrast-enhanced imaging data, and the use of contrast-enhanced imaging data would have lowered the number of patients included.

Given that English is not my native language, I have dedicated to express my thoughts and opinions in English to the best of my ability, and there may be some grammatical improprieties. I apologize for any language expression issues. If you have any questions, please do not hesitate to contact us.

In addition, we appreciate for Editors and Reviewers’ warm work earnestly, and hope that the response will meet with approval. Once again, thank you very much for your comments and suggestions.
