# Supplementary material for: Multi-institutional validation of a radiomics signature for identification of postoperative progression of soft tissue sarcoma
Source: Cancer Imaging. 2024 May 8;24:59. doi: 10.1186/s40644-024-00705-8 (PMC11077743; doi:10.1186/s40644-024-00705-8)
Supplement: Supplementary file 3 — Supplementary Material 3 [file 40644_2024_705_MOESM3_ESM.docx]

Dear Editors,

Thank you for your letter regarding our manuscript entitled " Multi-institutional Validation of a Radiomics Signature for Identification of Postoperative Progression of Soft Tissue Sarcoma" CAIG-D-23-00753.

Below is the updated copy of the manuscript that we have made available and have used red color to highlighting the revisions done from the previous round of review.

With regard to the extensive English Editing, we have used the English editing service.

**Acknowledgements**

We thank Michael Irvine, PhD, from Liwen Bianji (Edanz) (www.liwenbianji.cn) for editing the English text of a draft of this manuscript.

I apologize for any language expression issues. If you have any questions, please do not hesitate to contact us.

Thanks again for your time and letter.
